# Isocitrate dehydrogenase 1 mutation in cholangiocarcinoma impairs tumor progression by sensitizing cells to ferroptosis

**DOI:** 10.1515/med-2022-0477

**Published:** 2022-05-06

**Authors:** Li Su, Yi Huang, Lei Zheng, Zhifa Zhu, Yue Wu, Ping Li

**Affiliations:** Department of Integrated Traditional and Western Medicine in Oncology, The First Affiliated Hospital of Anhui Medical University, Hefei 230022, China; Center of Integrated Traditional and Western Medicine in Oncology, Anhui Medical University, Hefei 230022, China; Anhui University of Traditional Chinese Medicine, Hefei 230012, China; Department of Integrated Traditional and Western Medicine in Oncology, The First Affiliated Hospital of Anhui Medical University, No. 120, Wanshui Road, Hefei 230022, China

**Keywords:** cholangiocarcinoma, IDH1, mutation, ferroptosis

## Abstract

The present study intends to clarify the hypothesis that isocitrate dehydrogenase 1 (IDH1) mutation in cholangiocarcinoma impairs tumor progression by sensitizing cells to ferroptosis through the *in vitro* and *in vivo* experiments. Cholangiocarcinoma RBE cell line was transfected with IDH1 R132C mutation plasmids and treated with erastin to induce ferroptosis, which were then microscopically photographed. Cell viability rate was calculated by trypan blue staining. The lipid ROS level was determined by using flow cytometer. The BALB/c nude mice were injected subcutaneously with IDH1 knockout (KO), WT, or R132C mutation cell line, followed by injecting erastin intraperitoneally. The tumor tissue was surgically separated for the measurement of tumor volume and weight. The results showed that IDH1 mutant RBE cell line are sensitive to erastin-induced ferroptosis, evidenced by the increased number of propidium iodide-positive cells, the decreased cell viability, and increased lipid ROS level. However, current targeted inhibitors of IDH1 mutation (AG120 and IDH305) reversed these effects caused by IDH1 mutation. The *in vivo* experiment showed that IDH1 mutation in cholangiocarcinoma impairs tumor progression by sensitizing cells to erastin-induced ferroptosis. This study indicated that IDH1 mutation in cholangiocarcinoma impairs tumor progression by sensitizing cells to erastin-induced ferroptosis.

## 1 Introduction

Cholangiocarcinoma is an adenocarcinoma of the epithelial cells that occurs anywhere in the bile duct, which can be classified into intrahepatic, periportal, and distal types based on its clinical features. Surgery and liver transplantation are the recommended treatment options for all types of bile duct cancer in a small number of patients. The 5-year survival rate for patients with early-stage intrahepatic cholangiocarcinoma is 15%. However, if intrahepatic cholangiocarcinoma has spread to more distant parts of the body, the 5-year survival rate decreases to 2% [1]. Since surgery cannot completely remove metastatic tumors, other non-surgical treatments are recommended.

Iron-dependent lipid peroxide accumulation leads to cell death in the form of “ferroptosis” [2]. Recent studies have shown that ferroptosis is closely related to various human diseases and closely related to the mechanisms and treatments of tumorigenesis [3]. Studies have shown that herbal monomers (quercetin [4], artemisinin [5], salvia [6], etc.) can exert antitumor effects by inducing ferroptosis. In addition, Han et al. [7] found significantly lower levels of glutathione (GSH), peroxides, glutathione peroxidase (GPX) and ferrous iron [Fe^2+^] in extrahepatic cholangiocarcinoma compared to controls, suggesting the dysregulated iron metabolism and GPX-regulated ferroptosis.

Gene mutations are thought to play a key role in the development and progression of cholangiocarcinoma. Mutations in several genes (e.g., KRAS, Tp53, etc. [8]) have been found to be associated with the development of bile duct cancer. Mutations in isocitrate dehydrogenase 1 (IDH1) are presented in several human cancers including cholangiocarcinoma [9]. Wang et al. [10] found that mutated IDH1 was associated with a beneficial prognosis in cholangiocarcinoma and inhibited tumor growth by suppressing Akt signaling. Our previous study [11] demonstrated that IDH1 mutations in cholangiocarcinoma impair tumor progression by inhibiting isocitric acid metabolism. It raises the possibility that IDH1 mutation in cholangiocarcinoma impairs tumor progression by inducing ferroptosis. It was shown that the mutant form of IDH1/2 catalyzes the irreversible accumulation of 2-hydroxyglutaric acid (2HG), which is an alternative marker for IDH1 mutated intrahepatic cholangiocarcinoma [12], while 2HG produced by IDH1 mutations has been reported to promote erastin-induced ferroptosis in human fibroma cells and esophageal squamous carcinoma cells [13].

Erastin is a recognized inducer of ferroptosis that has been validated in a variety of tumor cells, such as breast cancer [14], hepatocellular carcinoma [15], and acute myeloid leukemia [16]. However, the effects of erastin on cholangiocarcinoma are little reported. Combined with the findings from previous studies, this study aimed to explore whether IDH1 mutation in cholangiocarcinoma impairs tumor progression by sensitizing cells to erastin-induced ferroptosis.

## 2 Materials and methods

### 2.1 Plasmid, chemicals, reagents, and materials

Cholangiocarcinoma RBE cell line was purchased from the American Type Culture Collection (ATCC). AG-120 (CSNpharm), IDH305 (DC Chemicals), erastin (MedChemExpress, ME) were purchased commercially. Male BALB/c nude mice were purchased from Guangzhou Laboratory Animal Center (Guangzhou, China).

### 2.2 Cell culture

Cells were cultured in complete Dulbecco’s modified Eagle medium (DMEM) containing fetal bovine serum (FBS, 10%) and antibiotics (penicillin and streptomycin, 1%) under 37°C in the presence of CO_2_ (5%) at constant humidity.

### 2.3 Cell viability

The cells (30 × 10^4^) were inoculated into 12 well plates and cultured under the specified treatment conditions (DMSO or erastin) for 12 h. Subsequently, the cells were digested with trypsin and collected for trypan blue staining. The cell viability was counted and calculated by using an automated cell counter (Count Star, IC 1000). Cell viability under test conditions is reported as a percentage relative to those before treatment.

### 2.4 Construction of IDH1 mutation cell line

The IDH1 knockout (IDH1 KO) cell line was constructed using CRISPR-Cas9 (Shanghai Liangtai Biotech Company, Shanghai, China). In brief, when the RBE cells reached 70% confluency, cells were transfected with CRISPR-Cas9 knockout plasmids containing guide RNA sequence of IDH1 and sequence of Cas9 protein.

The IDH1 R132C mutation cell line was constructed by transfecting the IDH1 KO cell line with IDH1 R132C mutation plasmids. IDH1 KO REB cells transfected with vector (empty plasmid) and plasmids containing WT IDH1 were respectively named as Vector group and IDH1 WT group.

### 2.5 Groups

After construction of IDH1 Mutation, IDH1 WT, and Vector cell line, cells were treated with 5 µM erastin (DMSO as control) for 12 h. In another experiment, after construction of IDH1 Mutation cell line, cells were treated with AG120 (0.5 µM) or IDH305 (5 nM) for 12 h, followed by administration of 5 µM erastin (DMSO as control) for 12 h.

### 2.6 Cell state and propidium iodide (PI) staining

According to the grouping, PI dye was added to the cells treated with erastin and incubated at 37°C for 15 min. The cell state was captured by microscope, and the PI-stained cells were observed and photographed by fluorescence microscope.

### 2.7 Cellular lipid ROS assay

Cells were seeded (6-well dishes, 400,000 cells/well) for 24 h, and then stained with C11-BODIPY 581/591 (Thermo Fisher Scientific, San Jose, CA, USA; D3861) for 30 min at 37°C and then harvested by trypsinization. Cells were re-suspended in PBS and strained through a 40 µm cell strainer (BD Falcon), and then analyzed cells using flow cytometer (Accuri C6, BD Biosciences) equipped with 488 nm laser for excitation. Data were collected from the FL1. A minimum of 10,000 cells were analyzed per condition.

### 2.8 Xenograft mouse model

Male BALB/c nude mice (6 weeks old) were housed in a room (a 12-h light–dark cycle) and fed under experimental conditions (22–24°C, humidity of 50 ± 5%). Animal procedures were approved by the Animal Care and Use Committee in First Affiliated Hospital of Anhui Medical University. After acclimatization for 1 week, the mice were grouped into three groups, which were, respectively, subcutaneously injected with 2 × 10^6^ REB IDH1 KO, REB IDH1 WT, or Vector cell line. After 1 week, mice in each group were sub-grouped into 2 groups: erastin was injected intraperitoneally at a dose of 15 mg/kg in one group, and an equal volume of DMSO was injected intraperitoneally in the other group, every 2 days for 20 days. Mice were administered once every 2 days, a total of 10 times. After the first administration, the diameter and volume of tumor tissue of mice in each group were measured every 3 days until the end of the administration. Animals were executed and tumor tissue was surgically isolated and removed, photographed, and focused. The tumor volume was measured every 3 days, according to the formula: length × width^2^ × 0.52.

### 2.9 Statistics

GraphPad 6.0 software was used for statistical analysis. Data were represented as mean value ± SD. Student’s *t*-test was used for the comparisons between the two groups and one-way analysis of variance (ANOVA) was used for the comparisons among three or more than three groups. *p* < 0.05 was considered as significant.

## 3 Results

### 3.1 IDH1 mutation promoted erastin-induced ferroptosis in cholangiocarcinoma RBE cell line

**Figure 1:**
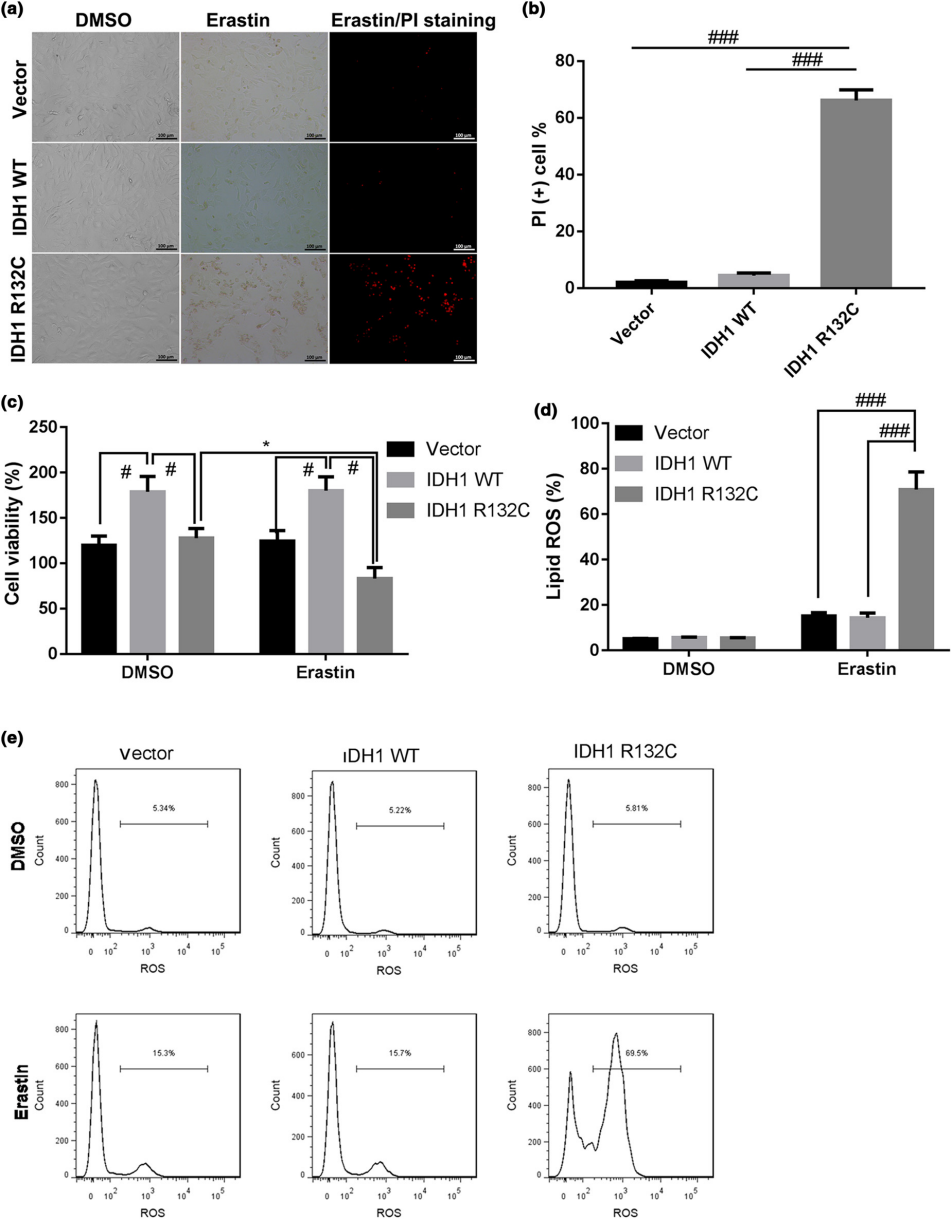
IDH1 mutation promotes erastin-induced ferroptosis in cholangiocarcinoma RBE cell line. The IDH1 KO RBE cell line was constructed using CRISPR-Cas9, and then the IDH1 KO cell line was, respectively, transfected with Vector, IDHI WT, or IDHI R132C mutation plasmid to construct the IDH1 WT or mutation cell lines, followed by erastin (5 μM) treatment for 12 h, and (a) cells were microscopically photographed. (b) The number of PI-positive cells was statistically quantified. (c) The trypan blue staining assay was used to determine the cell viability. (d and e) The lipid ROS levels were determined by using flow cytometer. *p < 0.05 vs DMSO treatment group, ^#^ *p* < 0.05, ^###^ *p* < 0.001 vs IDH1 WT or IDH1 R132C group.

To gain a clear understanding on the functional role of IDH1 in the ferroptosis, cholangiocarcinoma RBE cell line was, respectively, transfected with WT or Mutant *IDHI* expressing plasmid to construct and IDH1 WT or knockdown cell lines, followed by erastin treatment. The microscopical photograph showed that the number of PI-positive cells was enhanced in erastin-treated IDH1 WT cell line (Figure 1a and b). As shown in Figure 1c, the cell viability assay showed that the viability of erastin-treated IDH1 mutation cell line was significantly decreased as compared to that of erastin-treated IDH1 knockdown or IDH1 WT cell line. Besides, since excessive accumulation of lipid ROS is a critical cause of ferroptosis, the subsequent assay was performed to determine whether mutant IDH1 could promote the sensitivity of cholangiocarcinoma to erastin by increasing lipid ROS. As shown in Figure 1d–e, the lipid ROS levels in erastin-treated IDH1 mutation cell lines were increased compared to that in erastin-treated IDH1 knockdown or WT cell line. Taken together, IDH1 mutation could aggravate erastin-induced ferroptosis in cholangiocarcinoma by increasing lipid ROS.

### 3.2 Inhibitors of IDH1 mutation reversed its effects on erastin-induced ferroptosis in cholangiocarcinoma RBE cell line

**Figure 2:**
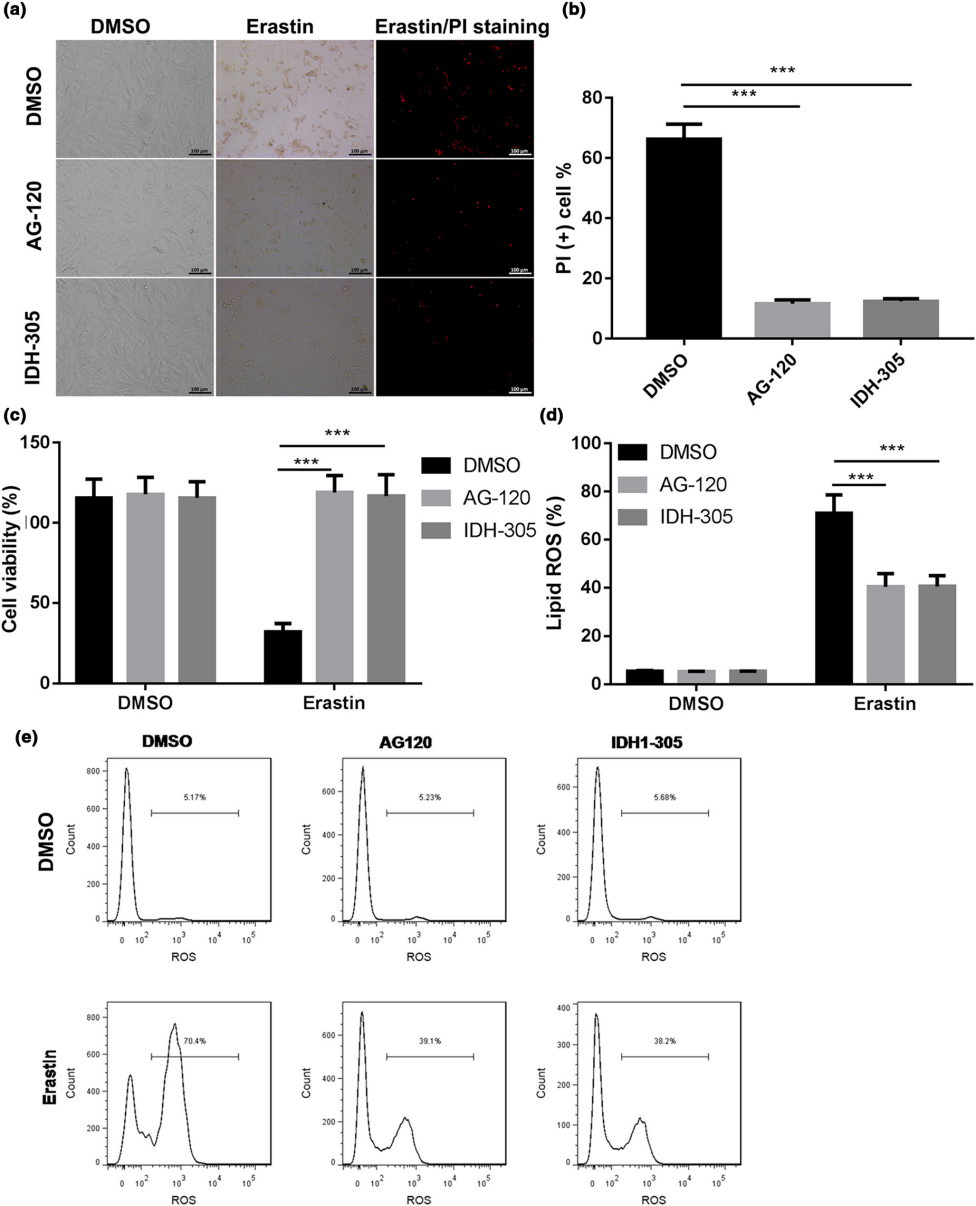
Inhibitors of IDH1 mutation reversed its effects on erastin-induced ferroptosis in cholangiocarcinoma RBE cell line. Current targeted inhibitors of IDH1 (AG120 and IDH305) were administrated into IDH1 R132C mutation cell line with erastin and DMSO treatment, and (a) cells were microscopically photographed. (b) The number of PI-positive cells was statistically quantified. (c) The trypan blue staining assay was used to determine the effects of AG120 or IDH305 on the cell viability. (d) The effects of AG120 or IDH305 on the lipid ROS levels were determined by using flow cytometer, and (d and e) statistically quantified. ***p < 0.001 vs DMSO treatment group.

Current targeted inhibitors (AG120 and IDH305) of IDH1 mutation can selectively inhibit mutant IDH protein, which were used to further verify the effects of IDH1 mutation on erastin-induced ferroptosis in cholangiocarcinoma. The microscopical photograph showed that, as compared to the DMSO treatment, the number of PI-positive cells was significantly decreased after AG120 or IDH305 treatment (Figure 2a and b). The Trypan blue staining assay showed that, compared to the DMSO treatment, the viability of IDH1 mutation cell line was significantly increased, but there was no significant change in the IDH1 mutation inhibitors groups (Figure 2c). Besides, as shown in Figure 2d and e, AG120 or IDH305 treatment decreased the lipid ROS levels in IDH1 mutation cell line as compared to that with DMSO treatment. Taken together, AG120 and IDH305 could reverse IDH1 mutation effects on erastin-induced ferroptosis in cholangiocarcinoma.

### 3.3 IDH1 mutation promotes erastin-induced tumor growth inhibition in cholangiocarcinoma

**Figure 3:**
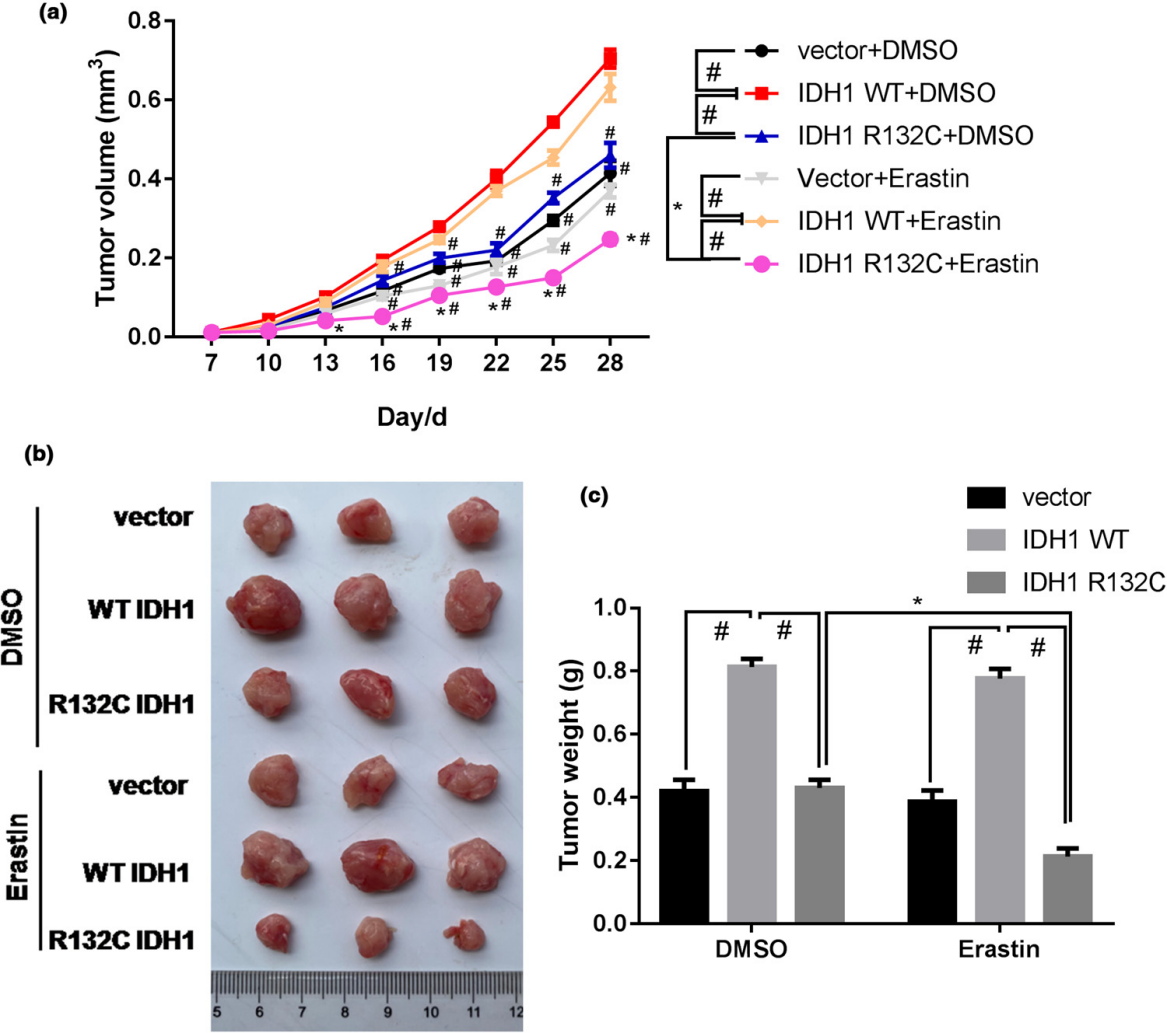
IDH1 mutation promotes erastin-induced tumor growth inhibition in cholangiocarcinoma. Male BALB/c nude mice were injected subcutaneously with IDH1 KO, WT, or mutation cell line. One week later, mice were injected intraperitoneally with erastin or DMSO at a dose of 15 mg/kg. (a) After administration, the diameter and volume of the tumor tissue were measured every 3 days until the end of the administration. The animals were executed and the tumor tissue was surgically removed for photography (b), and for the measurement of (c) tumor weight. *p < 0.05 vs DMSO treatment group, #*p* < 0.05 vs IDH1 WT group.

Male BALB/c nude mice were injected subcutaneously with IDH1 Knockdown cell line. One week later, the mice were injected intraperitoneally with erastin at a dose of 15 mg/kg. After the first administration, the diameter and volume of tumor tissue of mice in each group were measured every 3 days until the end of the administration. The animals were executed and the tumor tissue was surgically separated and removed for photography (Figure 3b). In total, the tumor volume and weight were, respectively, decreased by erastin treatment as compared to that by DMSO treatment in IDH1 mutation group, indicating the simulative effects of erastin on inhibiting tumor growth. Besides, erastin had no significant effect on IDH1 WT cell line and IDH1 knockout cell line. Taken together, IDH1 mutation promotes erastin-induced tumor growth inhibition in cholangiocarcinoma.

## 4 Discussion

In our previous study, IDH1 was identified as a high frequency mutated gene in patients with cholangiocarcinoma. The *in vitro* study showed that IDH1 promoted cell proliferation, invasion and migration, whereas conversed results were found in IDH1 R132C mutation cells. The *in vivo* study indicated that tumor volume in mice transplanted with the IDH WT cells was significantly increased when compared with that in mice transplanted with the IDH R132C mutation cells. These results supported that IDH1 R132C mutation impaired the development of cholangiocarcinoma [11]. Partly similar and more in-depth is this article reveals that IDH1 R132C mutation damages cholangiocarcinoma by inducing ferroptosis, and the evidence is the increase of lipid ROS level. However, current targeted inhibitors of IDH1 mutation (AG120 and IDH305) reversed these effects caused by IDH1 mutation. The *in vivo* experiment showed that IDH1 mutation promotes erastin-induced tumor growth inhibition in cholangiocarcinoma. Taken together, IDH1 mutation in cholangiocarcinoma impairs tumor progression by sensitizing the cells to erastin-induced ferroptosis.

IDH1 mutations have been identified in many types of cancer, including glioma [17], colon cancer [18], leukemia [19], and prostate cancer [20]. In recent years, ferroptosis has become an important subject of tumor research, whose predictive and prognostic value in patients with cholangiocarcinoma has been reported recently. By constructing ferroptosis scores, a previous study effectively predicted the prognoses of patients with cholangiocarcinoma, and discovered the potential gene targets to further enhance the efficacy of photodynamic therapy [21]. Besides, Han et al. [7] found significantly lower levels of GSH, peroxides, GPX, and ferrous iron [Fe^2+^] in extrahepatic cholangiocarcinoma compared to controls, suggesting dysregulated iron metabolism and GPX-regulated ferroptosis. This study indicated the important role of IDH1 mutation in the progression of ferroptosis in cholangiocarcinoma.

A previous study [7] indicated that mutant IDH1 reduces the protein level of GPX4, a key enzyme in removing lipid ROS and ferroptosis, and promotes depletion of glutathione. Consistently, the lipid ROS levels in erastin-treated IDH1 mutation cell line was increased compared to that in erastin-treated IDH1 knockout or WT cell line, indicating that IDH1 mutation could aggravate erastin-induced ferroptosis in cholangiocarcinoma by increasing lipid ROS. It is believed that excessive accumulation of lipid peroxide (lipid ROS) is a critical cause leading to ferroptosis [22]. Deeper investigations should be performed to further validate the links between lipid ROS and tumor progression in cholangiocarcinoma. Yuan et al. [23] indicated that targeting the ROS/Tnf/JNK axis may provide opportunities for intrahepatic cholangiocarcinoma therapy. Thongsom et al. [24] suggested that piperlongumine induces G2/M phase arrest and apoptosis in cholangiocarcinoma cells through the ROS-JNK-ERK signaling pathway.

Wandee et al. [25] indicated that derrischalcone suppresses cholangiocarcinoma cells through targeting ROS-mediated mitochondrial cell death, Akt/mTOR, and FAK pathways. The involvement of these pathways in the regulatory network between IDH1 mutation-mediated tumor progression and ROS-induced ferroptosis, need validations of more molecular mechanism experiments.

In conclusion, this study indicated that IDH1 mutation in cholangiocarcinoma impairs tumor progression by sensitizing cells to erastin-induced ferroptosis. This study may provide a potential mechanism for better understanding the role of IDH1 mutation in cholangiocarcinoma.
